# Co-transport of the nuclear-encoded *Cox7c* mRNA with mitochondria along axons occurs through a coding-region-dependent mechanism

**DOI:** 10.1242/jcs.259436

**Published:** 2022-08-16

**Authors:** Bar Cohen, Topaz Altman, Adi Golani-Armon, Anca F. Savulescu, Amjd Ibraheem, Musa M. Mhlanga, Eran Perlson, Yoav S. Arava

**Affiliations:** 1 Faculty of Biology, Technion – Israel Institute of Technology, Haifa 3200003, Israel; 2 Sackler Faculty of Medicine, Tel Aviv University, Tel Aviv 69978, Israel; 3 Faculty of Nanosciences and Nanoengineering, Technion – Israel Institute of Technology, Haifa 3200003, Israel; 4 Division of Chemical, Systems & Synthetic Biology, Department of Integrative Biomedical Sciences, Faculty of Health Sciences, Institute of Infectious Disease & Molecular Medicine, University of Cape Town, Cape Town 7925, South Africa; 5 Radboud Institute for Molecular Life Sciences (RIMLS), Radboud University Medical Center, 6525 GA Nijmegen, The Netherlands; 6 Epigenomics & Single Cell Biophysics Group, Department of Cell Biology, FNWI, Radboud University, 6525 GA Nijmegen, The Netherlands; 7 Department of Human Genetics, Radboud University Medical Center, 6525 GA Nijmegen, The Netherlands

**Keywords:** mRNA localization, Nuclear-encoded mitochondrial protein, Axonal transport

## Abstract

Nuclear-encoded mitochondrial protein mRNAs have been found to be localized and locally translated within neuronal processes. However, the mechanism of transport for those mRNAs to distal locations is not fully understood. Here, we describe axonal co-transport of *Cox7c* with mitochondria. Fractionation analysis and single-molecule fluorescence *in situ* hybridization (smFISH) assay revealed that endogenous mRNA encoding *Cox7c* was preferentially associated with mitochondria in a mouse neuronal cell line and within mouse primary motor neuron axons, whereas other mRNAs that do not encode mitochondrial protein were much less associated. Live-cell imaging of MS2-tagged *Cox7c* mRNA further confirmed the preferential colocalization and co-transport of *Cox7c* mRNA with mitochondria in motor neuron axons. Intriguingly, the coding region, rather than the 3′ untranslated region (UTR), was the key domain for the co-transport. Our results reveal that *Cox7c* mRNA can be transported with mitochondria along significant distances and that its coding region is a major recognition feature. This is consistent with the idea that mitochondria can play a vital role in spatial regulation of the axonal transcriptome at distant neuronal sites.

## Abstract (precis)

**Summary:** Biochemical and live imaging analyses show that in mouse axons, *Cox7c* mRNA is associated and transported with mitochondria. Mutational analysis identifies mRNA domains essential for co-transport.

## INTRODUCTION

Neurons extend axons over long distances and through various extracellular microenvironments, forming unique compartmentalized sites with specific functions and needs. These neuronal extensions largely rely on proper mitochondrial activity for their growth, function and survival. Indeed, mitochondrial dysfunction can lead to neurodegeneration and motor neuron diseases (Altman et al., 2021; Misgeld and Schwarz, 2017; Tan et al., 2014). Mitochondria also serve as important signaling hubs and are required for local protein synthesis events (Altman et al., 2021; Gershoni-Emek et al., 2018; Merrill and Strack, 2014; Rangaraju et al., 2019; Sheng, 2014; Spillane et al., 2013). However, how axonal mitochondria replenish their proteome or alter it in response to local needs is largely unknown. This question is particularly intriguing for distant mitochondria given that most of their proteins are derived from nucleus-transcribed mRNAs, yet the duration of protein and mitochondria transport from the soma is prolonged (Maday et al., 2014; Terenzio et al., 2017; Twelvetrees et al., 2012). These mitochondria are likely replenished by the local synthesis of proteins given that mRNAs encoding mitochondrial proteins have been identified at distant axonal sites and local synthesis appears necessary for mitochondrial function (Altman et al., 2021; Cioni et al., 2019; Harbauer et al., 2022; Lee et al., 2022). For local translation to occur, mRNAs need to be localized to axons, as has been demonstrated for most nuclear-encoded mitochondrial proteins (Aschrafi et al., 2010; Cioni et al., 2019; Farias et al., 2020; Maciel et al., 2018; Minis et al., 2014; Rotem et al., 2017).

An accepted model for transporting mRNAs to neuronal extensions involves the formation of RNA granules that shuttle by interactions with kinesin motors and RNA-binding proteins at their 3′ untranslated region (UTR) (Kanai et al., 2004; Mofatteh and Bullock, 2017; Sahoo et al., 2018). Recently, an endosome-mediated mechanism for protein replenishment was suggested, in which mRNAs encoding mitochondrial proteins are transported to distant sites through their association with Rab7-containing endosomes. These mRNAs are then locally translated, and the protein is targeted to nearby mitochondria (Cioni et al., 2019).

Previous data demonstrating that mRNAs are associated with mitochondria (Eliyahu et al., 2012; Lesnik et al., 2014; Marc et al., 2002; Vardi-Oknin and Arava, 2019; Williams et al., 2014), and that miRNAs are associated with axonal mitochondria (Gershoni-Emek et al., 2018) led us to investigate the possibility of a mitochondria-based axonal transport mechanism for specific mRNAs. We found significant association of the cytochrome *c* oxidase subunit 7C (*Cox7c*) mRNA (a component of complex IV of the respiratory chain) with mitochondria from a neuronal cell line and primary motor neurons. MS2-tagging and live imaging of *Cox7c* mRNA revealed significant cotransport with mitochondria. Intriguingly, the coding region showed a much more prominent impact on localization and cotransport than the 3′ UTR.

## RESULTS AND DISCUSSION

### Mitochondrial association of endogenous *Cox7c* mRNA in motor neuron axons

**Fig. 1.**
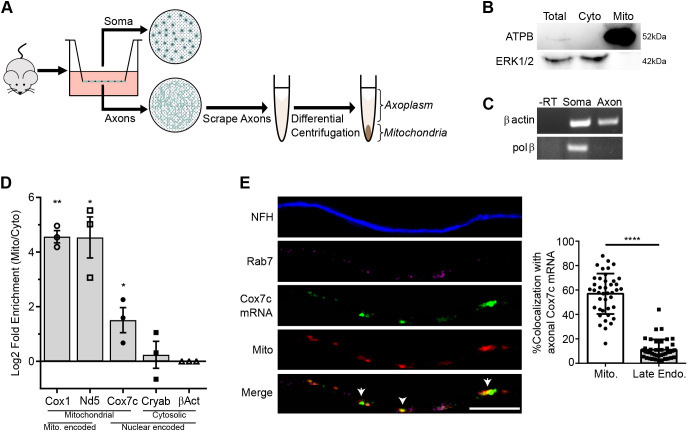
**Nuclear encoded mitochondrial gene Cox7c mRNA is associated with mitochondria in motor neuron axons.** (A) Schematic workflow used for membrane-based compartmental isolation of primary motor neuron axons. (B) Western blot analysis for fractionated axonal samples, with a mitochondrial marker (ATPB) and cytosolic marker (ERK1/2) demonstrating purity of mitochondria samples. (C) RT-PCR analysis for soma-specific mRNA (Pol β) confirming separation purity. β-actin represents mRNA present in both fractions. -RT, without reverse transcriptase. Images in B and C representative of three independent biological repeats. (D) RT-qPCR analysis was performed on axonal RNA samples from mitochondrial and axoplasm fractions for mRNAs encoded in the mitochondria (*Cox1* and *Nd5*) or the nucleus (*Cox7c*, *Cryab* and β-actin). All values are normalized to β-actin transcript levels. Error bars are s.e.m.; *n*=3 independent biological repeats. *P<0.05, **P<0.01 (two-way ANOVA with Holm–Sidak correction). (E) Left, representative images of smFISH performed on primary motor neurons for *Cox7c* mRNA (green) along with immunostaining for axons by neurofilament heavy chain (NFH, blue), late endosomes (Rab7 marker, magenta) and mitochondria (MitoTracker staining, red). Arrows indicate areas of colocalization between mitochondria and *Cox7c* mRNA. Scale bar: 10 µm. Right, colocalization analysis of *Cox7c* mRNA with mitochondria and late endosomes signals. Error bars are s.d.; *n=*41 axons from three repeats. ****P<0.0001 (unpaired two-sided *t*-test).

To examine the possibility that the mRNA encoding mitochondrial protein Cox7c is co-transported with mitochondria, we first performed a biochemical analysis to determine the association of its mRNA with mitochondria. We used a fractionation approach of differential centrifugation (Eliyahu et al., 2011) to purify the mitochondria from primary mouse motor neuron axons and N2a cells (Fig. 1A; Fig. S1A). To separate neuronal axons from the cell bodies, motor neurons were grown on a porous membrane (‘modified Boyden chamber’) (Gershoni-Emek et al., 2018; Rotem et al., 2017). The mitochondria fraction purity was verified by western blot analysis, revealing an enrichment of mitochondrial proteins and absence of cytosolic markers (Fig. 1B; Fig. S1B). RT-PCR analysis confirmed the absence of soma-marker mRNA (*Polb*, encoding Pol β) from the axonal fractions; however, a known axonal mRNA (*Actb*, encoding β-actin) was clearly detected in axonal fractions (Fig. 1C). Furthermore, quantitative RT-PCR (RT-qPCR) for mRNAs transcribed inside the mitochondria, NADH dehydrogenase, subunit 5 (*Nd5*) and cytochrome *c* oxidase I (*Cox1*), revealed enrichment of these mRNAs in the mitochondrial fraction (Fig. 1D; Fig. S1C). Next, we analyzed *Cox7c* mRNA, which encodes a mitochondrial protein transcribed in the nuclear genome. Our choice of this mRNA was based on previous studies showing enrichment of mRNAs encoding components of the oxidative phosphorylation pathway near mitochondria (Williams et al., 2014). Importantly, *Cox7c* mRNA has also been found enriched in rodent axons (Aschrafi et al., 2010, 2016; Natera-Naranjo et al., 2012; Rotem et al., 2017). Indeed, *Cox7c* mRNA appeared to be enriched in the mitochondrial fraction compared to other known axonal localized mRNAs that do not encode mitochondrial proteins such as *Cryab* (a crystallin B chain) and β-actin (Fig. 1D; Fig. S1D). *Cryab* still had a significant association with mitochondria, so it might represent ‘hitchhiker’ mRNAs as its quantity in the cytosolic fraction appeared at higher levels than in the mitochondrial fraction.

Next, we performed single-molecule fluorescence *in situ* hybridization (smFISH) to visualize *Cox7c* in primary motor neuron axons and N2a cells together with mitochondrial staining. Colocalization analysis revealed extensive colocalization of up to 60% of *Cox7c* mRNA with mitochondria, compared with a weak association to other axonal organelles, namely endosomes, marked with the late-endosomal marker Rab7 (Fig. 1E; Fig. S1E). Interestingly, analysis of colocalization at the soma revealed similar enrichment near mitochondria compared to endosomes (Fig. S2). This suggests that preferred mitochondrial association of *Cox7c* is initiated at the cell body and maintained throughout the axon.

### *Cox7c* mRNA is co-transported with mitochondria in motor neuron axons

**Fig. 2.**
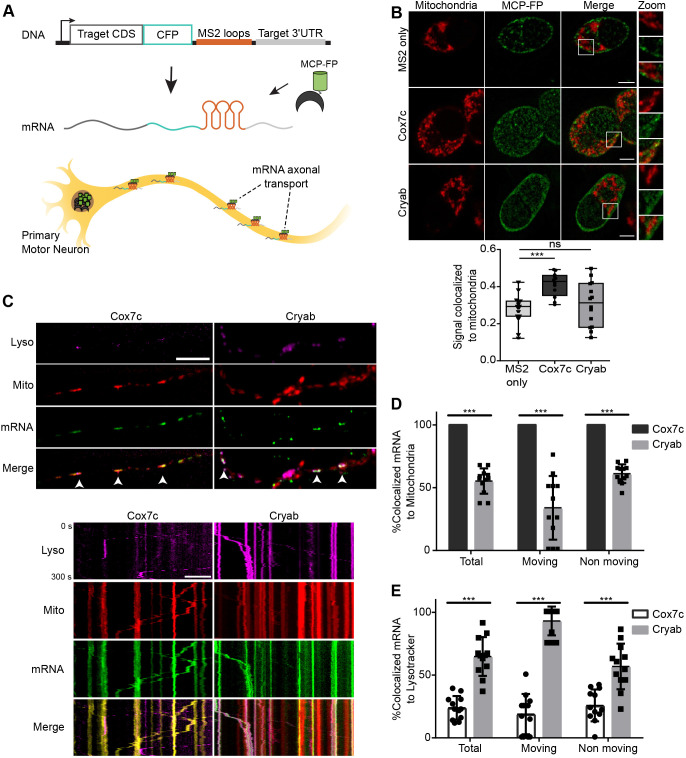
**Live imaging reveals cotransport of mRNA with mitochondria.** (A) Scheme of mRNA imaging system. Top, schematic representation of the genetic constructs encoding target coding sequence (CDS) and the 3′ UTR of *Cox7c* and *Cryab* genes, which were cloned with multiple MS2 aptamers and CFP. Bottom, scheme of mRNA imaging in primary motor neurons. MS2-loop-containing constructs are introduced into motor neurons together with a plasmid expressing fluorescent protein-tagged MS2-binding protein (MCP-FP). Cells are grown in microfluidic chambers and signals are quantified along axonal extensions. (B) Top, representative images of MS2–mRNA constructs expressed in N2a cells together with a vector expressing MS2-binding protein fused to YFP (MCP–FP; green) colocalized with mitochondria staining (red). Scale bars: 5 μm. Bottom, quantification of the mRNA signal overlapping with mitochondria. Each data point represents a frame. The box represents the 25–75th percentiles, and the median is indicated. The whiskers show the minimum and maximum values; *n*=19, 20, 20 cells from 15, 12, 14 frames for MS2 only, *Cox7c* and *Cryab*, respectively. n.s (non-significant) *P*>0.05, ***P<0.001 (Mann–Whitney test). (C) Representative images (top) and kymographs (bottom) of primary motor neurons infected with viral vectors expressing MCP–GFP and *Cox7c* or *Cryab* MS2–mRNA constructs (green), showing colocalization with axonal mitochondria (Mitotracker, red) and acidic compartments (Lysotracker, magenta). Arrowheads indicate colocalization of mRNA and mitochondria. Scale bars: 10 μm. (D) Quantification of axonal cotransport between mRNA and the mitochondria separated to moving and static mRNA. Error bars are s.d.; *n*=12 and 12 axons for *Cox7c* and *Cryab*, respectively, from three independent biological cultures. ***P<0.001 (two-way ANOVA with Holm–Sidak correction). (E) Quantification of axonal cotransport between mRNA and acidic compartments separated to moving and static mRNA. Error bars are s.d.; *n*=12 and 12 axons for *Cox7c* and *Cryab*, respectively, from three biological independent cultures. ***P<0.001 (two-way ANOVA with Holm–Sidak correction).

To explore the possibility for cotransport of *Cox7c* mRNA with mitochondria, we utilized the MS2 system (Wu et al., 2012) to tag candidate mRNAs and follow their position and transport. The complete coding region (CDS) of *Cox7c* and *Cryab* (as a low association, control marker) were cloned in-frame to CFP in an MS2-loop-bearing expression vector (Fig. 2A). Furthermore, the 3′ UTR of each gene was cloned downstream of the MS2 loops. This yielded transcripts (designated ‘full’) containing the entire transcript of each gene tagged with multiple MS2 loops. Each construct was transfected into N2a cells together with a plasmid expressing MS2-binding protein fused to YFP (MCP–YFP). Confocal microscopy was used to measure colocalization with MitoTracker Red-stained mitochondria (Fig. 2B). Quantification of mRNA signals that colocalized with mitochondrial signals revealed lower mitochondrial colocalization of both MS2 control vector and *Cryab*-expressing MS2 construct compared to the *Cox7c*-expressing MS2 construct (Fig. 2B), consistent with the behavior of the endogenous mRNAs (Fig. 1).

Next, we introduced the plasmids with MS2-tagged genes and MCP–GFP by viral infection into primary motor neurons and performed live imaging microscopy with mitochondria and lysosomal markers to capture their movement with high spatial and temporal resolution (Fig. 2A,C). Notably, all detected signals for *Cox7c* appeared to colocalize with mitochondria (100% colocalization), whereas less than 50% of *Cryab* signals were colocalized. Furthermore, all moving *Cox7c* mRNA signals were in complete concordance with mitochondria movement, whereas only a small fraction of *Cryab* movements were in concordance with moving mitochondria (Fig. 2D,E; Movies 1,2). In contrast to *Cox7c* localization, *Cryab* mRNA showed a high degree of lysosomal co-transport, suggesting that mitochondria binding is primarily pivotal for *Cox7c* axonal transport whereas *Cryab* is dependent on acidic compartments for localization (Fig. 2C,E). Thus, mRNA encoding *Cox7c* is associated with axonal mitochondria and appears to be transported with them along the axon.

### The coding region contains key determinants for *Cox7c* cotransport

**Fig. 3.**
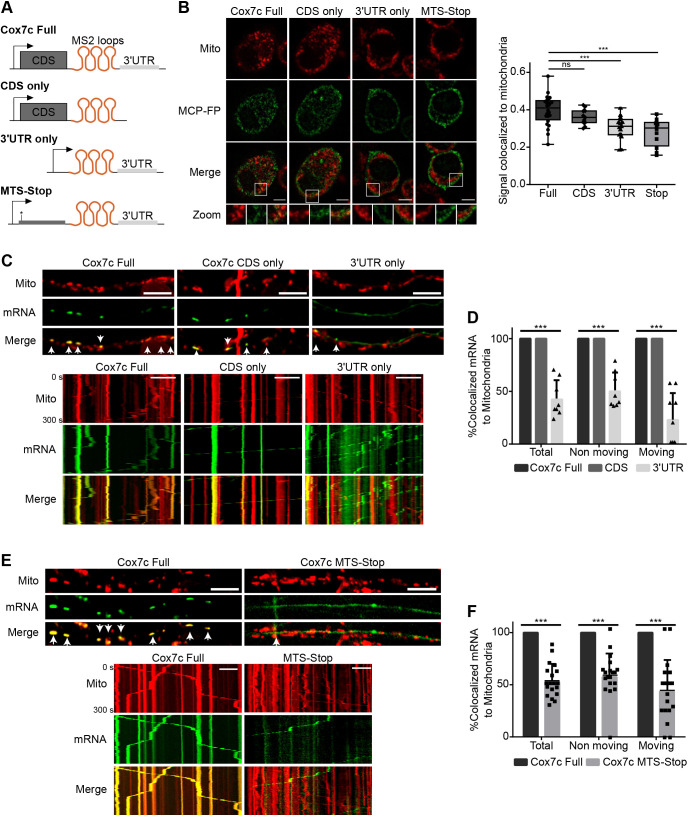
**CDS determinants are important for Cox7c axonal mitochondrial association and cotransport.** (A) Schematic representation of MS2-loops *Cox7c* Full, *Cox7c* CDS only, *Cox7c* 3′ UTR only or Cox7c MTS-Stop (a point mutation generating a stop codon closely downstream of the initiation codon) constructs. (B) Left, representative images of N2a cells transfected with the MS2 constructs with MS2 binding protein fused to YFP (MCP-FP; green). Scale bars: 5 μm. Right: quantification of the mRNA signal overlapping with mitochondria. Each data point represents a frame. The box represents the 25–75th percentiles, and the median is indicated. The whiskers show the minimum and maximum values; *n*=42, 17, 25, 18 cells from 24, 11, 15, 12 frames for Cox7c Full, CDS only, 3′ UTR and MTS-Stop, respectively. n.s. (non-significant) *P*>0.05, ***P<0.001 (Mann–Whitney test). (C) Representative images (top) and kymographs (bottom) of primary motor neurons infected with viral vectors expressing *Cox7c* Full, *Cox7c* CDS only or *Cox7c* 3′ UTR only and MCP–GFP (green). Axons show axonal colocalization with mitochondria (red) in the Full and CDS only but not in the 3′ UTR only construct. Arrowheads indicate colocalization of mitochondria and mRNA signals. Scale bars: 10 μm. (D) Quantification of axonal cotransport of mRNA and mitochondria, separated according to moving and static mRNA. Error bars are s.d.; *n*=8, 12, 8 axons from three biological independent cultures. ***P<0.001 (two-way ANOVA with Holm–Sidak correction). (E) Representative images and kymographs of primary motor neurons infected with viral vectors expressing Full Cox7c or Cox7c ‘STOP’ and MCP–GFP (green). Axons show axonal colocalization with mitochondria (red) in the Full *Cox7c* but not in the *Cox7c* ‘STOP’ construct. Arrowheads indicate colocalization of mitochondria and mRNA signals. Scale bars: 10 μm. (F) Quantification of axonal cotransport of mRNA and mitochondria, separated according to moving and static mRNA. Error bars are s.d.; *n*=9 and 19 axons from three biological independent cultures. ***P<0.001 (two-way ANOVA with Holm–Sidak correction).

The classical model for axonal localization of mRNA suggests that domains within the 5′ and 3′ UTR sequence direct mRNA into axons, mainly through serving as binding sites for RNA-binding proteins (Kanai et al., 2004; Lee et al., 2022; Rotem et al., 2017; Sahoo et al., 2018; Turner-Bridger et al., 2020). To identify which mRNA regions are important for cotransport of Cox7c with mitochondria, either the *Cox7c* coding region (CDS) alone or the 3′ UTR alone were cloned into an MS2-loop-bearing expression vector. Northern blot and RT-qPCR analyses confirmed that all transcripts (Full, CDS only or 3′ UTR only) are of the expected length (Fig. S3A), and their expression levels are similar (Fig. S3B). To determine the localization of these mRNA variants, N2a cells were transfected with these constructs and with the MCP–YFP plasmid. Colocalization analysis revealed that whereas the mRNA carrying the CDS region exhibited similar colocalization values to those of the entire *Cox7c* mRNA (‘Full’), the mRNA carrying only the 3′ UTR region had a significantly lower association with mitochondria (Fig. 3A,B). This indicates that the coding region is sufficient to induce mitochondrial localization. Moreover, inserting a mutation that generates a stop codon in the coding sequence, specifically within the mitochondria-targeting signal (MTS), which is important for mRNA localization (Eliyahu et al., 2010; Lesnik et al., 2014), yielded a similar outcome and decreased mitochondrial localization of the mRNA (Fig. 3A,B). Thus, our data indicate that the *Cox7c* coding region has a stronger impact on mRNA localization to the mitochondria than its 3′ UTR.

Finally, we introduced the deletion variants into primary motor neurons (Fig. 3C,D; Movies 3,4). Also here, the CDS-only colocalization signals appeared very similar to those of the full construct. Furthermore, focusing on the motile mRNAs in axons revealed that all these transcripts were moving together with mitochondria. However, this was not the case for the 3′ UTR variant; most of its signal did not correlate with the movement of mitochondria. Furthermore, analysis of the CDS mutated construct (MTS-stop) revealed lower mitochondrial localization (Fig. 3E,F; Movie 5).

The data presented here reveal a unique mode of mRNA transport in neurons. We propose a model in which the nuclear-encoded mitochondrial mRNA *Cox7c* is associated and transported with axonal mitochondria. We describe new determinants, namely elements within the coding region, that are important for proper mRNA association and hence cotransport with mitochondria. Although the inserted mutation (a change to a stop codon) might suggest involvement of translating ribosomes in this process, this possibility is yet to be explored. We previously reported a similar colocalization mode for mRNAs encoding mitochondrial proteins with mitochondria in yeast cells (Eliyahu et al., 2010, 2012; Lesnik et al., 2014), thus suggesting a conserved mechanism.

Other mRNA elements that mediate mitochondria localization are usually located in 3′ UTRs (Glock et al., 2017; Mofatteh and Bullock, 2017; Sahoo et al., 2018; Schatton and Rugarli, 2018). Indeed, a cis-element located within the 3′ UTR of another Cox-family member (CoxIV, the rat homolog of mouse *Cox4i1*) has been shown to mediate its axonal localization (Aschrafi et al., 2010; Kar et al., 2014). Sequence analysis and secondary structure predictions did not reveal such a motif in *Cox7c*. Nevertheless, the mRNA containing only the *Cox7c* 3′ UTR was found to have some association with mitochondria, and disruption of the MTS did not fully abolish axonal cotransport. Thus, multiple, not necessarily mutually exclusive, mRNA regions might ensure mRNA association with mitochondria.

Recently, Harbauer et al. have found that *PINK1* mRNA is co-transported with mitochondria in rodent neurons, and described the role of RNA-binding proteins in this process. Those interactions have a role in local translation of PINK1, which in turn supports mitochondria quality at distant sites (Harbauer et al., 2022). Taken together with the data presented herein, we suggest that mitochondrial association with various mRNAs encoding mitochondria proteins is a broad phenomenon. Furthermore, mitochondria might serve as a shuttle vector for diverse types of mRNA to neuronal extremities, and by that, spatiotemporally regulate the cellular proteome. Another open question at this stage is whether the interaction of mRNA with mitochondria occurs already at the cell body, and whether this association is maintained throughout the lifetime of the mRNA. The enriched association of mRNA with mitochondria at the soma (Fig. S2) suggests that this is the case. However, higher resolution temporal and spatial tools are needed to follow mRNA traffic along the entire neuron.

A study in retinal ganglion cells of *Xenopus* revealed an endosome-mediated mode of transport for two mRNAs (*lamnb* and *vdac2*) with roles in mitochondrial function (Cioni et al., 2019) and a transcriptome-wide analysis in *Ustilago maydis* suggested that many more mitochondrial mRNAs are transported by endosomes (Olgeiser et al., 2019). Also herein we found at least partial colocalization of *Cox7c* mRNA with endosomal markers and acidic compartments. We therefore cannot exclude the possibility that some *Cox7c* transcripts are transported with endosomes and handed out to mitochondria at the destination. Moreover, transport through a classical ribonucleoprotein (RNP)–motor protein-mediated mechanism also cannot be excluded. Thus, multiple pathways might work in parallel to ensure sufficient protein content for mitochondria at sites distant from the cell body (Müntjes et al., 2021). Notably, lysosomes have been found to efficiently shuttle a non-mitochondrial mRNA (β-actin) in axons (Liao et al., 2019). Thus, mRNA trafficking by moving organelles emerges as a general concept in the neuronal transport of diverse mRNA types.

## MATERIALS AND METHODS

### Animals

The HB9::GFP (stock no. 005029) mouse colony was originally obtained from Jackson Laboratories. The colony was maintained by breeding with ICR mice (Jackson Laboratories). Mice were genotyped by DNA extraction and PCR using a KAPA ReadyMix kit (Thermo Fisher Scientific) kit. For experimental purposes, only wild-type (WT) embryos of pregnant mice (GFP negative) were taken for motor neuron cultures. Animal experiments were performed under the supervision and approval of the Tel-Aviv University Committee for Animal Ethics.

### Cell growth, transfection and treatments

Mouse neuroblastoma cells (N2a), a kind gift from Prof. Shai Berlin, Technion, Haifa, Israel, were cultured in Dulbecco's modified Eagle's medium (DMEM; Sigma D5796) supplemented with 10% fetal bovine serum (FBS; Biological Industries, 04-001), 2% penicillin-streptomycin (Biological Industries, 03-031) and 2 mM L-glutamine (Biological Industries, 03-020), at 37°C in a 5% CO_2_ atmosphere. Cells were split every 2–3 days by trypsinization (Biological Industries, 03-052). Cells were routinely tested for mycoplasma contamination. For transfection experiments, cells were grown to 60% confluency and transfected using jetPRIME reagent (Polyplus, 11415) according to the manufacturer's instructions. Protein synthesis inhibition treatments were performed by incubating the cells with 200 μg/ml puromycin (Sigma-Aldrich) in growth medium for 45 min before imaging.

### Primary motor neuron culture

Motor neurons were cultured from embryonic day (E)12.5 ICR mouse embryos as previously described (Gershoni-Emek et al., 2018). Briefly, spinal cords were dissected, incubated with trypsin for 10 min, and triturated. The neuron-containing ‘soup’ was collected through a cushion of bovine serum albumin (BSA; Sigma-Aldrich), and the pellet was resuspended and centrifuged through a 10.4% Optiprep (Sigma-Aldrich) gradient for 20 min at 760 ***g*** with the brake turned off. The cells were collected from the interface, centrifuged through a cushion of BSA, counted and plated. Motor neurons were grown and maintained at 37°C 5% CO_2_ incubator, in complete neurobasal medium (Gibco) containing 2% B27 (Thermo Fisher Scientific), 2% horse serum (Biological Industries), 25 μM β-mercaptoethanol, 1% penicillin-streptomycin (Biological Industries) and 1% Glutamax (Gibco) supplemented with 1 ng/ml GDNF, 0.5 ng/ml CNTF, and 1 ng/ml BDNF (Alomone Labs).

### Lentiviral production and infection

Lentiviral constructs were produced in HEK cells (kindly provided by Prof. Eran Bachrach, Tel Aviv University, Israel) using a second-generation packaging system with Gag-Pol and VSVG helpers. HEK cells were maintained in Dulbecco’s minimum essential medium (DMEM) supplemented with 1% GlutaMAX, 10% fetal bovine serum, and 1% penicillin-streptomycin on a 100-mm dish at high confluence. For lentiviral preparation, medium was replaced with antibiotic-free medium and transfected with 10 µg of MS2 vector, 10 µg of MCP-GFP and 20 µg of helper plasmids (see below for plasmid details). Plasmids were placed in a calcium phosphate transfection mix (25 mM HEPES, 5 mM KCl, 140 mM NaCl, and 0.75 mM Na_2_PO_4_ with 125 mM CaCl_2_) in 1 ml volume per plate. The medium was replaced 8 h after transfection, and supernatants were harvested 2 days afterwards. Lentiviral particles were concentrated 10-fold by using the PEG virus precipitation kit (Abcam); samples were mixed 1:7 with PEG, incubated overnight at 4°C and centrifuged at 10,000 ***g*** for 30 min. Samples were kept at −80°C until use. Viral infection was performed using 10–20 µl of lentivirus per microfluidic chamber.

### Mitochondria fractionation from N2a cells

At 24 h post transfection, N2a cells were detached and washed with ice-cold phosphate-buffered saline (PBS), and pelleted by centrifugation (600 ***g*** for 5 min at 4°C). The cell pellet was resuspended in homogenization buffer [HM; 0.6 M mannitol, 50 mM Tris-HCl pH 7.4, 5 mM MgAc, 100 mM KCl, 1 mM DTT, 1 g/l BSA, 200 μg/ml cycloheximide (CHX), 1 mM PMSF, 1 μM leupeptin, 1 μM pepstatin, 0.3 μM aprotinin and 0.04 U/μl RiboLock RNase Inhibitor (Thermo Fisher Scientific)], and lysed by 15–20 strokes of a Dounce homogenizer. Lysate was centrifuged twice (1000 ***g*** for 10 min at 4°C) to pellet intact cells and nuclei, and supernatant (designated ‘Total’) was further centrifuged at 15,000 ***g*** for 15 min at 4°C to obtain a cytosolic fraction (supernatant) and a mitochondrial fraction (pellet).

### Axonal harvesting and mitochondrial fractionation

Porous membranes (‘modified Boyden chamber’) with 1 μm pores were placed in a six-well culture plate (Corning). The membranes were coated with poly-DL-ornithine (1.5 μg/ml in PBS) overnight at 37°C then with laminin (3 μg/ml in double-distilled water; Sigma-Aldrich) for at least 2 h at 37°C prior to motor neuron plating. Primary motor neurons were then plated on the membranes (10^6^ neurons per a membrane insert) and grown with complete neurobasal medium. After 10 days *in vitro* (DIV), membranes were washed three times with warm PBS, followed by gentle scraping of the axonal (bottom) part of the membrane into a tube containing mitochondria HM [0.6 M mannitol, 50 mM Tris-HCl pH 7.4, 5 mM MgAc, 100 mM KCl, 1 g/l BSA, 200 μg/ml CHX, 1 mM DDT, 1× protease inhibitor cocktail (Roche) and 0.04 U/μl RiboLock RNase Inhibitor]. Four membranes were collected for each experimental repeat. Axons collected in HM were sub-fractionated to cytosolic (supernatant, Cyto) and mitochondrial (pellet, Mito) fractions by two cycles of 12,000 ***g*** centrifugation for 15 min at 4°C.

### Western blot analysis

Samples from N2a cells and primary motor neurons were lysed and fractionated in mitochondria HM (or immediately lysed after axonal harvest for somatic fraction), and were boiled in mixed in 1:4 (v/v) 5× Laemmli sample buffer for 5 min at 95°C. Next, samples were loaded on polyacrylamide gel and blotted on nitrocellulose membrane. The membrane was blocked with 5% BSA (N2a samples) or 5% milk (primary motor neuron samples) in TBS with 0.1% Tween 20 for 1 h. Membranes of N2a cells samples were incubated for 1 h at room temperature (RT) with primary antibodies for ATP5A (1:1000, Abcam Ab119688), GAPDH (1:2000, Abcam Ab181602) or GFP (1:3000, Aves labs AB_2307313). The membranes were next incubated with secondary antibodies against mouse-IgG (1:20,000, Sigma A5906), rabbit-IgG (1:20,000, Sigma A9169) or chicken-IgG (1:10,000, Sigma-Aldrich AP162P) for 45 min at RT and were exposed to ECL imager after 5 min incubation with EZ-ECL reagent (Biological Industries). Membranes of primary motor neuron samples were incubated overnight at 4°C with primary antibodies for ERK1/2 (1:10,000, Sigma Aldrich M5670) and ATPB (1:1000, Abcam ab14730). The membranes were next incubated with secondary anti-HRP antibody (1:10,000, Jackson Laboratories Cat. 715-035-151) for 2 h at RT and were exposed to ECL imager after 5 min incubation with ECL reagent (Thermo Fisher). Full images for western blots shown in this paper are given in Fig. S4.

### RT-qPCR

RNA was extracted from all samples (fractionated cells or axons) using TRIzol reagent according to the manufacturer's protocol (Thermo Fisher Scientific). RNA was reverse transcribed using Maxima First Strand cDNA Synthesis kit (Thermo Fisher Scientific). To verify fraction purity, cDNA was subjected to PCR amplification with β-actin and Polymerase β primers followed by gel electrophoresis. Quantification of target genes was performed by real-time qPCR using SYBR green (Thermo Scientific). Targets were normalized to β-actin, and log_2_ fold-change between mitochondrial and cytosolic fractions was calculated (ΔΔCt). All RT-qPCR primers are listed in Table S1.

### smFISH

Design and manufacture of RNA FISH probes for use in the smFISH for N2a cells were performed according to the protocol by (Raj et al., 2008). Multiple 20-mer oligonucleotide probes conjugated to dyes, targeting the mRNAs *Rnr1*, *Cryab* and *Cox7c* were purchased (Biosearch Technologies). The *Rnr1* probes were conjugated to TAMRA dye and the *Cryab* and *Cox7c* probes were conjugated to Quasar-670 dye. N2a cells were fixed in 3.7% formaldehyde for 15 min at 37°C followed by washes in PBS and overnight permeabilization in 70% Ethanol at 4°C. Cells were rehydrated in wash buffer [10% formaldehyde, 2× saline-sodium citrate buffer (SSC)] for 5 min. Hybridization was conducted overnight in a humidified chamber at 37°C in hybridization buffer [10% dextran sulfate, 1 μg/μl *E. coli* tRNA, 2 mM vanadyl ribonucleoside complex, 0.02% RNase-free BSA, 10% formamide, 2× SSC] combined with 50 ng of the desired RNA probe. Cells were then washed three times (each wash 30 min at room temperature) with FISH wash buffer (10% formaldehyde, 2× SSC). Cells were then incubated in equilibration buffer (0.4% glucose, 2× SSC) for 5 min and counter stained with 1 μg/ml DAPI (Life Technologies). Coverslips were mounted in imaging buffer (3.7 μg/μl glucose oxidase and 1 U catalase in equilibration buffer) and imaged on a StellarVision microscope using Synthetic Aperture Optics (SAO).

Labeling of single mRNA molecules in mouse motor neurons was performed by smiFISH as previously described (Tsanov et al., 2016). Briefly, primary motor neurons were grown for 7 days on 13 mm coverslips. Labeling procedures were performed in an RNase-free environment. Cultures were fixed with 4% PFA and permeabilized overnight with 70% ethanol. Samples were incubated with SSC (Sigma) based 15% formamide (Thermo Fisher Scientific) buffer for 15 min. Samples were hybridized overnight at 37°C with 16 FLAP-Y-Cy-3-tagged complementary oligonucleotide probes, targeting regions in *Cox7c* mRNA (IDT) in hybridization mix [15% formamide, 1.7% tRNA (Sigma; R1753), 2% FLAP:Probe mix, 1% VRC (Sigma), 1% BSA (Roche), 20% dextran sulfate (Sigma; D8906), 1× SSC]. Samples were washed twice with warm 15% formamide in 1×SSC buffer (1 h each), then 30 min with 1× SSC buffer, and 30 min with 1× PBS. Prior to immunostaining samples were washed with Tris-HCl pH 7.5, 0.15 M NaCl buffer, and then permeabilized with same buffer supplemented with 0.1% Triton X-100. Samples were blocked by 2% BSA (in the same buffer) for 30 min. Samples were incubated with primary chicken anti-neurofilament heavy chain (NFH; 1:1000, ab72996 Abcam) and mouse anti-Rab7 (1:200, ab50533 Abcam) antibodies in blocking buffer overnight at 4°C, and then with fluorescent secondary antibodies in blocking buffer for 2 h at room temperature. Samples were mounted with Vectashield Antifade reagent.

### Plasmid construction

All mouse CDS and 3′ UTR used were amplified from mouse cDNA using the primers indicated in Table S2. Fragments were cloned into phage-cmv-cfp-24xms2 vector (Addgene plasmid #40651) under the control of CMV promoter and upstream of the woodchuck hepatitis virus post-transcriptional regulatory element (WPRE). CDS fragments (from the start codon to the penultimate codon) were inserted in frame, upstream of CFP at the AgeI and NotI sites. 3′ UTR fragments were inserted downstream to the MS2 stem loops cassette at the ClaI site. Constructs designated as ‘Full’ contain both the CDS and 3′ UTR of the respective gene. ‘CDS’ or ‘3′ UTR’ designations indicate constructs with only these regions of the gene (cloned at the above restriction sites) and the CFP reporter. MTS-stop is similar to Full with an insertion of a nucleotide at the fifth codon, which yields a stop codon. Correct construction was confirmed by sequencing and Correct transcript length was validated by northern blotting. We note that all constructs contained 12 MS loops instead of the expected 24, presumably due to recombination during cloning.

The MCP–YFP plasmid was a gift from Prof. Yaron Shav-Tal (Bar Ilan University, Ramat Gan, Israel). The MCP–GFP plasmid used for axonal live-imaging was phage-ubc-nls-ha-tdMCP-gfp (Addgene plasmid #40649).

### Motor neuron axonal transport in microfluidic chambers

Polydimethylsiloxane (PDMS) microfluidic chambers (MFC) were made in-house and were cast as previously described (Ionescu et al., 2019). To enable motor neuron plating and medium exchange, four 6-mm wells were punched in the end of each channel surrounding the microfluidic grooves. The microfluidic devices were then cleaned, first with adhesive tape to remove dirt and then sterilized in 70% ethanol for 10 min. The microfluidic devices were then dried, and UV irradiated for 10 min, followed by attachment to a 35 mm FluoroDish glass-bottom dish. For primary motor neuron plating, 150,000 neurons were concentrated and seeded in 4 µl medium to allow adhesion, and, after 45 min 25 ng/ml BDNF-enriched complete neurobasal medium was added. Lentiviral constructs were added to the proximal compartment at 2 h post plating. From DIV 2, a neurotrophic and volume gradient was maintained between the distal and proximal compartment to encourage axonal crossing. Neurons were grown for between 6 and 8 days to allow axonal crossing and lentiviral expression and were then stained with 100 nM MitoTracker Deep-Red FM and 100 nM Lysotracker Red (Thermo Fisher Scientific) for 30 min at 37°C followed by three washes with warm medium. For live axonal transport, movies of 100 frames were collected at 3 s intervals from the distal part of the MFC grooves. The movies were obtained using a Nikon Ti microscope equipped with a Yokogawa CSU X-1 spinning disc and an Andor iXon897 EMCCD camera controlled by Andor IQ3 software and 60× oil objective.

Axonal transport colocalization analysis was performed as previously described (Altman et al., 2019; Gershoni-Emek et al., 2018). Briefly, transport movies were analyzed through kymograph generation, using the Kymo ToolBox ImageJ plugin. Colocalization was determined where a mRNA particle kymograph track overlapped with a track in the corresponding mitochondria kymograph. Moving particles were defined after movement of more than 10 µm in a specific direction during the 5-min duration.

### Live imaging and analysis of N2a cells

N2a cells were seeded in 24-well glass-bottom plates at a density of 50,000 cells/well and allowed to grow for 24 h. Cells were then transfected with MCP–YFP plasmid and one of the MS2 plasmids (0.25 μg of each). At 24 h after transfection, mitochondria were stained with 100 nM MitoTracker Red CMXRos (Thermo Fisher Scientific) for 30 min at 37°C and washed with PBS before confocal live imaging. Images were captured using the LSM 710 inverted confocal microscope (Zeiss) with 63×1.4 NA oil immersion objective lens. *Z*-stack images were acquired with 0.5 μm *z*-step size and image pixel size of 0.1 μm.

mRNA signal detection and mitochondria colocalization quantification were performed using Imaris software. Mitochondria signal was masked and segmented using the built-in surface algorithm in Imaris. mRNA spots of 0.5 μm diameter were detected using the built-in spot detecting function. To take into account high signal variability between cells and to ensure correct signal detection, thresholds were chosen manually. A spot was considered colocalized if at least half of it overlapped with the mitochondria surface detected. Image processing for display proposes was performed using FIJI software. To filter background noise, the MCP channel was subjected to the FTT bandpass filter and background subtraction. The mitochondrial signal channel was subjected to background subtraction.

### Statistical analysis

Statistical analysis was performed using build in tools in Microsoft Excel or GraphPad Prism 6, with specific tests indicated in each figure legend.

## Supplementary Material

Supplementary information
