# Supplementary material for: Co-transport of the nuclear-encoded Cox7c mRNA with mitochondria along axons occurs through a coding-region-dependent mechanism
Source: J Cell Sci. 2022 Aug 16;135(16):jcs259436. doi: 10.1242/jcs.259436 (PMC9481926; doi:10.1242/jcs.259436)
Supplement: Supplementary information [file joces-135-259436-s1.pdf]

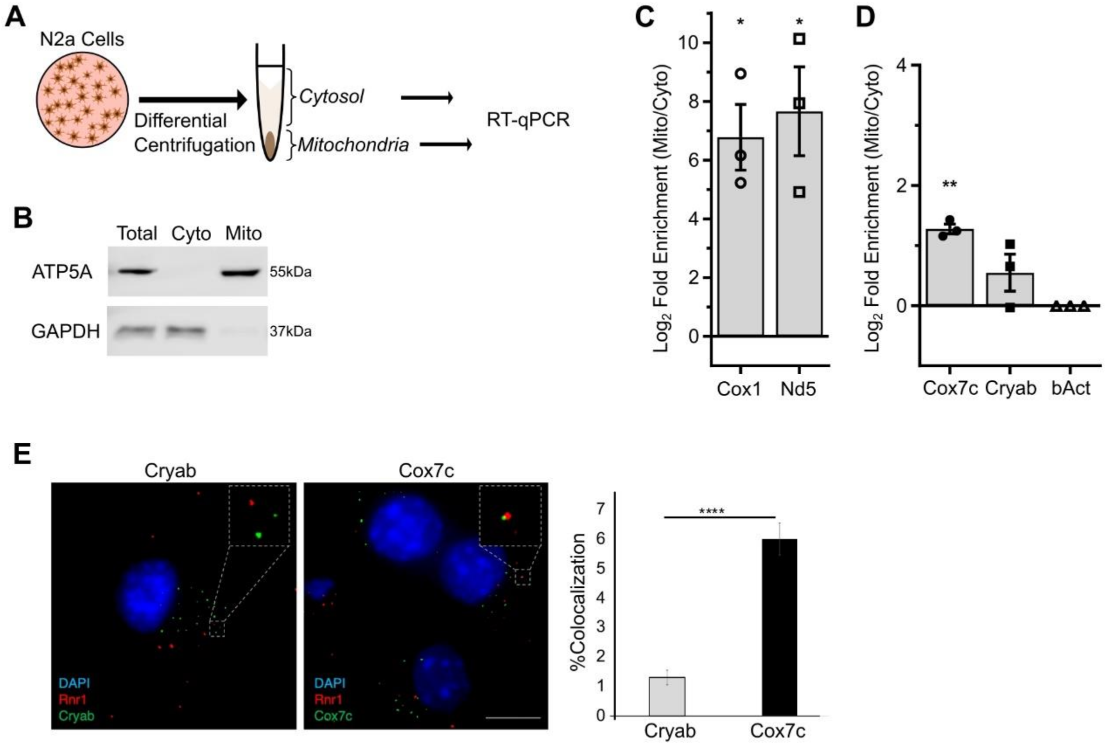


**Fig. S1. Cox7c mRNA is associated with mitochondria from N2a cells.** A) Schematic workflow: N2a cells were collected and fractionated by differential centrifugation into cytosolic and mitochondrial fractions followed by RNA purification and RT-qPCR. B) Western analysis of the unfractionated total sample, cytosolic (Cyto) and mitochondrial (Mito) fractions with a mitochondria marker (ATP5A) and cytosol marker (GAPDH), demonstrating fractionation purity. C) RT-qPCR analysis of mitochondrial and cytosolic fraction for mRNAs encoded inside the mitochondria. All values are normalized to β-actin transcript levels and presented as log2 fold enrichment of mitochondria and cytosolic Ct signals. Error bars are SEM. Two-way ANOVA, *$p<0.05$. n = 3 independent biological repeats. D) RT-qPCR analysis of mitochondrial and cytosolic fraction for mRNAs encoded in the nucleus, separated to those that their proteins are destined to mitochondria or cytosol. Quantification as in panel C. Error bars are SEM. Two-way ANOVA, **$p<0.01$. n = 3 independent biological repeats. E) Representative images of smFISH done on N2a cells with the mRNA (green) of Cryab (left) or Cox7c (right). Probes targeting the 21S mitochondrial rRNA (Rnr1-red) were used to detect mitochondria signal. The histogram presents the percent of spots of each mRNA that colocalize with Rnr1 signals. Student's t-test, ****$p<0.0001$. n=82, 69 cells for Cox7c and Cryab, respectfully, in three independent experiments. To minimize ambiguity, only spots with overlapping signals were considered as colocalized. Scale bar = 5 µm.

Journal of Cell Science • Supplementary information

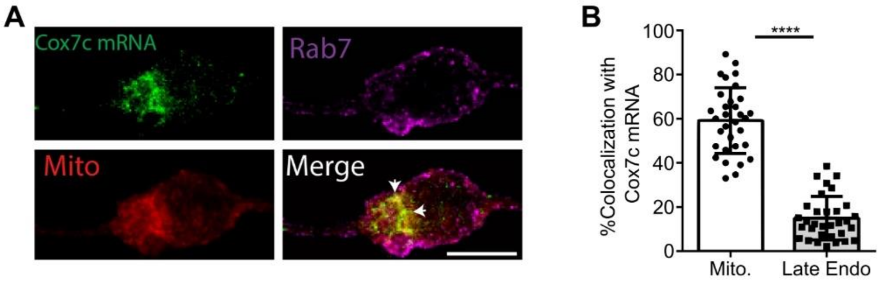


**Fig. S2. Cox7c mRNA is associated with mitochondria in motor neurons cell body.** A) Representative images of smFISH done on primary motor neurons for Cox7c mRNA (green) along with immunostaining for late endosomes (Rab7 marker, magenta) and mitochondria staining (MitoTracker, red). Arrows indicate areas of colocalization between mitochondria and Cox7c mRNA. Scale bar =10μm. B) Colocalization analysis of Cox7c mRNA with mitochondria and late endosomes signals. n=33 cells from 3 repeats. Error bars are SD. Unpaired-t-test ****p<0.0001.

Journal of Cell Science • Supplementary information

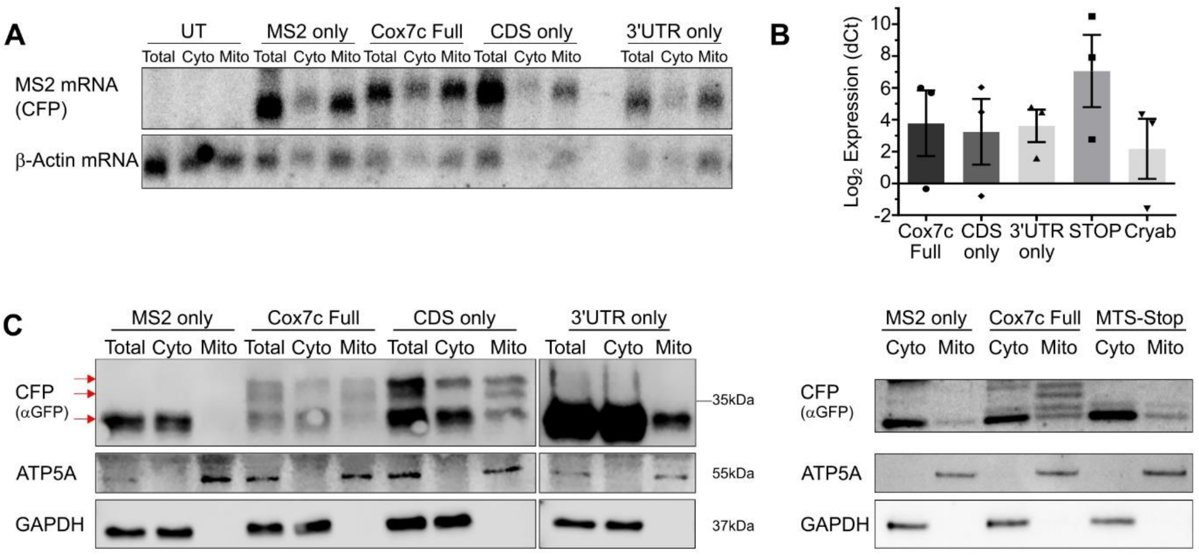


**Fig. S3. MS2 constructs are expressed in N2a cells.** A) Northern analysis to verify transcripts at the expected size, in unfractionated total sample (Total), cytosolic (Cyto) and mitochondrial (Mito) N2a fractions either from untransfected cells (UT) or transfected with the indicated constructs. Probes indicating MS2 transcripts expression (CFP) and endogenous mRNA expression (β-Actin) were used. B) RT-qPCR analysis of total MS2 mRNAs levels, in N2a cells transfected with different MS2 constructs. CFP levels are used as indicators for mRNA levels. All values are normalized to β-actin transcript and presented as log2 expression of Ct signals. Error bars are SEM. n = 3 independent biological repeats. C) Western blot analysis of cytosolic (Cyto) and mitochondrial (Mito) fractions from cells transfected with the indicated constructs, to verify translation of the Cox7c-CFP fusion proteins. Mitochondria marker (ATP5A) and cytosol marker (GAPDH), demonstrating fractionation sufficiency. Upper, middle and lower red arrows to the left, indicate the precursor Cox7c-CFP, mature Cox7c-CFP and CFP alone, respectively.

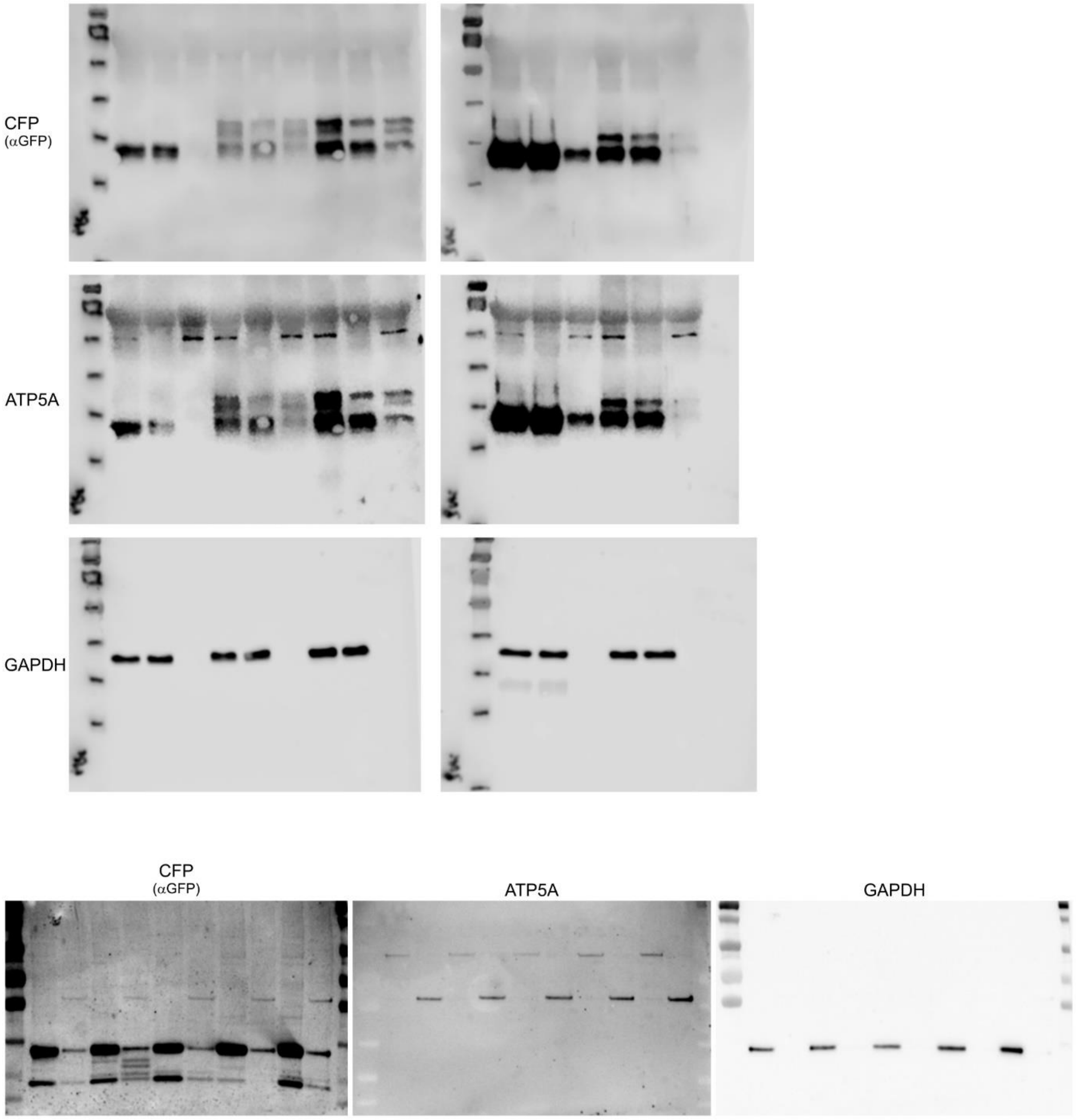


**Fig. S4. Blot transparency.** Full, uncropped western blots corresponding to western blots in figure S3 C.

Journal of Cell Science • Supplementary information

## Table S1. List of primers used for real-time PCR

| Primer name | Primer sequence |
|---|---|
| β-actin Fw | ATGGATGACGATATCGCTG |
| β-actin Rv | GTTGGTAACAATGCCATGTTC |
| Cox1 Fw | TCCAACTCATCCCTTGACATC |
| Cox1 Rv | TCCTGCTATGATAGCAAACACT |
| Cox7c Fw | AGCATGTTGGGCCAGAGT |
| Cox7c Rv | ACTGAAAACGGCAAATTCTT |
| Cryab Fw | ACTTCCCTGAGCCCCTTCTA |
| Cryab Rv | TGCTTCACGTCCAGATTCAC |
| Nd5 Fw | AACCACACCTAGCATTCCTAC |
| Nd5 Rv | CAGGCGTTGGTGTTGCAGGTA |
| CFP Fw | CGTGACCACCCTGACCTGG |
| CFP Rv | TCCTGGACGTAGCCTTCGG |

## Table S2. List of primers used for plasmid cloning

| Primer name | Primer sequence |
|---|---|
| Cox7c CDS Fw | ATATGCGGCCGCATGTTGGGCCAGAGTAT |
| Cox7c CDS Rv | CAGCACCGGTCGTTTTTTAAGTAGCTGGTGT |
| Cox7c 3’ UTR Fw | CCCGATCGATGGATATTTAATTCATCCCT |
| Cox7c 3’ UTR Rv | GCCGATCGATCAGAGACGAGGCATTG |
| Cox7c CDS no-MTS Fw | ATATGCGGCCGCCACCATGGTTAGCCACTATGAGGAG |
| Cox7c CDS stop-MTS Rv | ATATGCGGCCGCCACCATGGTTTTGGGCTAGAGTAT |
| Cryab CDS Fw | ATTTGCGGCCGCATGGACATCGCCATCC |
| Cryab CDS Rv | CAATACCGGTACCTTCTTAGGGGCTGCG |
| Cryab 3’ UTR Fw | CCCGATCGATATCCCCTTTCCTCATTG |
| Cryab 3’ UTR Rv | GCCGATCGATCAGAGACGAGGCATTG |

Journal of Cell Science • Supplementary information

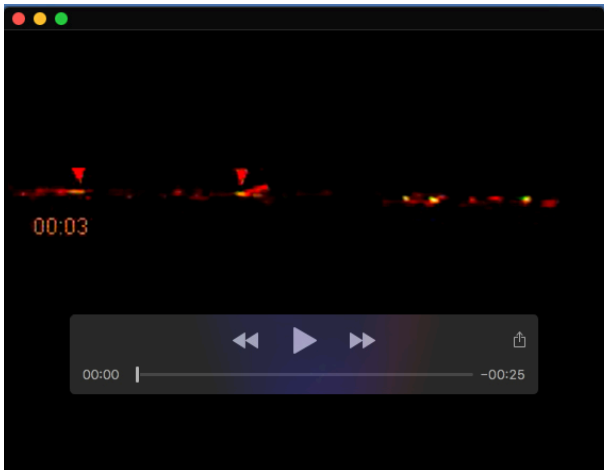


**Movie 1.** mRNA of Cox7c-MS2 visualized with MCP-GFP construct and displays complete co-localization and co-transport with mitochondria in axons. Live-cell imaging of mRNA particles (green) together with mitotracker marked mitochondria (red) in a motor neuron axon. The red arrows indicate Cox7c mRNA particles co-transported with mitochondria. Movie speed is 10 frames per second, at 3 seconds per frame.

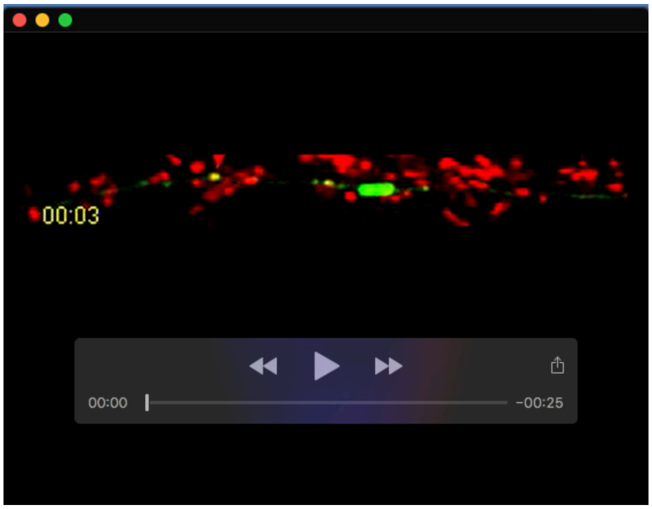


**Movie 2.** mRNA of Cryab-MS2 visualized with MCP-GFP construct and displays low co-localization and co-transport with mitochondria in axons. live-cell imaging of mRNA particles (green) together with mitotracker marked mitochondria (red) in a motor neuron axon. The red arrows indicate Cryab mRNA particles colocalized with static mitochondria. Note that moving mRNA puncta are not colocalized with mitochondria. Movie speed is 10 frames per second, at 3 seconds per frame.

Journal of Cell Science • Supplementary information

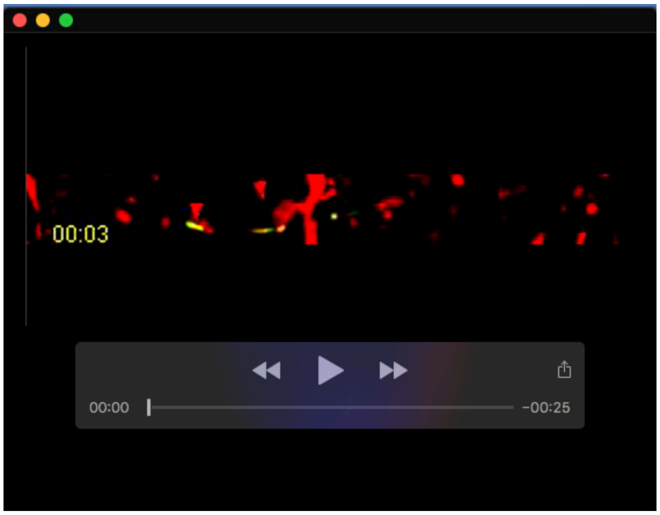


**Movie 3.** mRNA of Cox7c-CDS-only MS2 visualized with MCP-GFP construct displays complete co-localization and co-transport with mitochondria in axons. Live-cell imaging of mRNA particles (green) together with mitotracker-marked mitochondria (red) in a motor neuron axon. The red arrows indicate Cox7c CDS-only mRNA particles co-localized with static mitochondria and co-transported with moving mitochondria. Movie speed is 10 frames per second, at 3 seconds per frame.

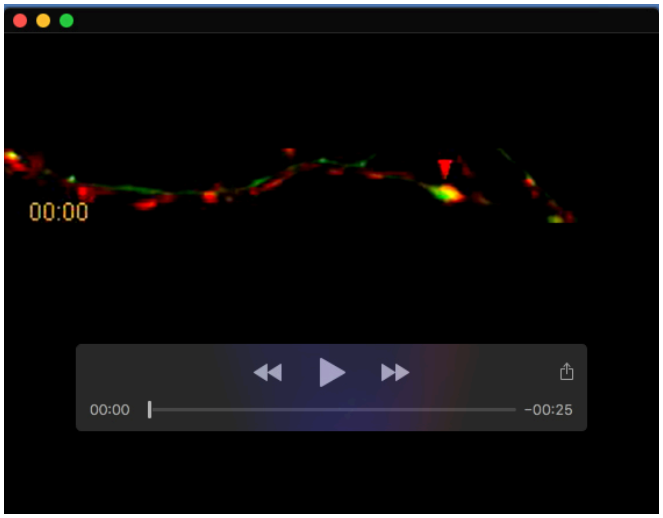


**Movie 4.** mRNA of Cox7c 3'UTR-only MS2 visualized with MCP-GFP construct and displays low frequency of co-localization with mitochondria in axons. Live-cell imaging of mRNA particles (green) together with mitotracker-marked mitochondria (red) in a motor neuron axon. The red arrows indicate Cox7c 3'UTR-only mRNA particles co-localized with static mitochondria. Note that moving mRNA puncta are not co-localized with mitochondria. Movie speed is 10 frames per second, at 3 seconds per frame.

Journal of Cell Science • Supplementary information

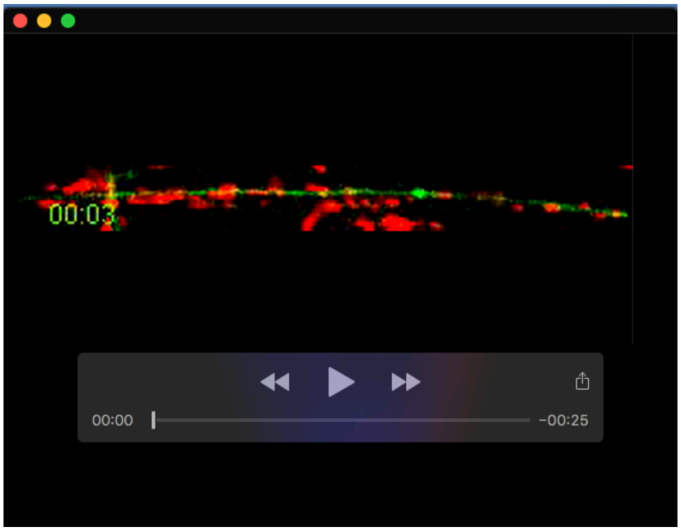


**Movie 5.** mRNA of Cox7c-MTS STOP MS2 visualized with MCP-GFP construct and displays low frequency of co-localization with mitochondria in axons. Live-cell imaging of mRNA particles (green) together with mitotracker-marked mitochondria (red) in a motor neuron axon. The red arrow indicates Cox7c-MTS-STOP mRNA particles co-localizes with static mitochondria. Note that moving mRNA puncta are not colocalized with mitochondria. Movie speed is 10 frames per second, at 3 seconds per frame.

Journal of Cell Science • Supplementary information
